# Malaria Vaccine Trial Results Are Negative, but Important

**DOI:** 10.1371/journal.pmed.0010047

**Published:** 2004-10-26

A malaria vaccine called ME-TRAP, which targets the pre-erythrocytic stage of the disease, was not effective at reducing natural infection rates in semi-immune African adults, according to the report of a randomized controlled trial published this month in *PLoS Medicine*. “This first field efficacy trial was an important milestone in the progression of new recombinant vectored vaccines to deployable products,” says Adrian Hill (University of Oxford, United Kingdom), the lead investigator of the study. “The safety profile was excellent and the efficacy data provide a first indication of the levels of cellular immunogenicity that will be required for preventing infection,” he says.

Hill and his co-workers used a heterologous prime–boost vaccination technique. They gave the volunteers two vaccines—a DNA priming vaccine followed by a modified vaccinia virus Ankara (MVA) that acted as a booster. The DNA and MVA vaccines both had the same insert coding for thrombospondin-related adhesion protein (TRAP; a pre-erythrocytic antigen) and a string of T cell epitopes (called ME for “multiple epitopes”).

Hill's team had previously shown that ME-TRAP vaccines given in prime–boost sequence could induce large T cell responses in healthy volunteers from the UK and could delay parasitemia in a sporozoite challenge test (Nat Med 9: 729–735). The next step was to do a randomized controlled trial in Gambia to determine whether this vaccination strategy could provide protection against natural Plasmodium falciparum infection.

The researchers recruited volunteers from 13 Gambian villages that were close to the alluvial flood plain and so were at high risk of developing malaria. They randomly assigned the 372 volunteers to receive either two doses of the DNA ME-TRAP vaccine followed by a single dose of MVA ME-TRAP, or three doses of rabies vaccine. This three-dose schedule is similar to the one used by the World Health Organization/United Nations Children's Fund Expanded Program on Immunization. Two weeks before the third dose was given, all the volunteers received antimalarial drugs to clear blood-stage P. falciparum infections.

The time to first infection, the primary end point of the study, was similar in the two groups, with an estimated vaccine efficacy of only 10%. However, the effector T cell response to the TRAP antigen T9/96, measured one week after the third vaccination, was 80 times higher in the DNA/MVA vaccine group than in the rabies vaccine group.

“It is absolutely crucial that results like these are published, since the failures, as well as the successes, need to be documented if we are to move towards rational strategies for optimizing malaria vaccines,” says Tom Smith from the Swiss Tropical Institute, who was not involved in the study. “At the same time, it makes sense to move on quickly without shedding too many tears, in a field that is moving much faster than it was before the recent injections of money from the Gates Foundation, but where it is still impossible to second-guess the results of field trials. This is partly because we do not have any good proxy measures of effective immunity in P. falciparum, and partly because this is a fertile area for trying out new techniques, such as DNA vaccines, where there is still a lot to learn.”

Hill is planning to do further trials that address the important question of whether this type of vaccine can prevent the symptoms of malaria. “The next step,” says Hill, “is to assess newer vaccine regimes that employ two viral vectors rather than DNA and to study prevention of malaria rather than infection.”

